# Measuring outcomes in self-harm trials: what is important and what is achievable?

**DOI:** 10.1192/bjo.2020.5

**Published:** 2020-02-12

**Authors:** Allan House

**Affiliations:** Professor of Liaison Psychiatry, Leeds Institute of Health Sciences, University of Leeds, UK. Email: a.o.house@leeds.ac.uk

**Keywords:** Outcome studies, psychosocial interventions, deliberate self-harm

## Abstract

There are a number of candidates as useful outcomes in self-harm research – repetition of self-harm; symptom states; quality of life, social participation. Repetition of self-harm has been the predominant choice of researchers, not least because of its status as a risk for eventual suicide. Use of alternatives would respond to the preferences of study participants, but there are substantial methodological constraints. Nonetheless more attention could be given to the use of outcomes other than repetition. Another option is to broaden the approach to evaluation design, incorporating advances in the use of observational data either alone or linked to data from trials.

There is no shortage of randomised controlled trials (RCTs) that evaluate the effect of an intervention after an episode of self-harm: the latest systematic reviews in the Cochrane Library include 55 studies involving adult participants^1^ and 11 trials involving children and adolescents.^2^ Even so, uncertainty remains about the best therapeutic action to take; there are methodological weaknesses in the primary research and many trials are simply too small to resolve uncertainties. More research is therefore needed. In the UK neither of the major supporters of trials in mental health (the UK's National Institute for Health Research Health Technology Assessment programme and the Medical Research Council) has funded a substantial trial in this area for more than 20 years; as we press for more and more substantial research into self-harm interventions the study by Owens and colleagues in this issue^3^ raises an important question: are we certain that we know what to measure as the main outcomes in our trials?

## Important outcomes: the main candidates

To date, the answer to this question has overwhelmingly been: repetition of self-harm. Thus, in a recent systematic review Hetrick and colleagues^4^ were able to include 36/45 (80%) of identified trials in an analysis of the effect of intervention on repetition – typically within 6 or 12 months of an index episode. It is not difficult to understand why this is, as common sense points us towards responding to the immediate indication for intervention – self-harm, and repetition of self-harm, is the biggest known risk for eventual suicide.^5^ Hospital attendance after self-harm, which is typically caused by a further episode, is the main driver of healthcare cost and reducing such attendances is therefore the main determinant of cost-effectiveness of an intervention.

Repetition of self-harm as a primary outcome in trials is not without its problems. Most people who present for healthcare after self-harm do so by going to a hospital for treatment for a recent act, and this is where most trials recruit their participants. In the hospital-presenting population, repetition, although common, is still the exception – 75–80% of participants will not repeat in the next 12 months.^5^ Repetition of self-harm as a primary outcome will therefore not apply to the majority of trial participants. Apart from other considerations this means that trials have to recruit large numbers of participants to demonstrate benefit, with unpalatable cost implications for research funders. A second consideration is that it is not easy to collect complete information on repetition. Records of hospital attendance are reliable,^6^ especially in the era of electronic records, but many episodes of self-harm do not lead to hospital attendance. The alternative, self-reported repetition even when the act does not lead to healthcare, is unreliable. Finally, reduction of repetition may not be an immediate concern of trial participants – a caveat applies, particularly to those with a history of multiple repetition, where the act can serve adaptive or coping functions that it may be jeopardous to abandon before robust change in other areas is achieved.

What other outcome measures might we use in self-harm trials? The candidates fall into three broad categories: symptom states; function, and especially social function; and quality-of-life measures, which are often (at least to some extent) composites of the other two.

The most widely measured symptoms in this context are mood related. Self-report scales can be used to record, for example, depressive symptoms with or without recourse to associated diagnostic statements. Other more cognitive experiences, such as hopeless thinking or ideas about suicide, are obviously relevant to the self-harm population. Indeed, they are commonly used in self-harm trials, usually as secondary outcomes.^7^ One drawback is that although they may be associated with subsequent repetition or suicide, the link is not clear cut and their predictive value is moderate. Another weakness is that such symptom states are less clearly related to healthcare use and therefore less relatable to the cost-effectiveness of an intervention.

Quality-of-life measures such as the EQ-5D^8^ and the SF36^9^ can be applied to the whole population but, because of their origins in research in physical medicine, are often too oriented to physical aspects of life experience. Others such as CORE-OM^10^ are essentially measures of symptom state. The advantage of quality-of-life measures is that if they are preference-based they allow derivation of quality-adjusted life-years and thereby allow estimation of the comparative cost-effectiveness of an intervention. It is uncertain how they relate to other outcomes or predict repetition or suicide.

Functional measures have a different aim, which is to pick up on people's ability to live a life that they find satisfactory – by being able to do everything they want physically, by being able to socialise or see others as much as they want, and by being able to seek help and support as and when they need it. In addition to the evidence of participant preference pointed out by Owens and colleagues, there are theoretical reasons why social participation might be important, because of the likelihood that social disconnectedness and its subjective equivalent (what has been called thwarted belongingness^11^) may be a significant risk for eventual suicide. For the purpose of measuring functional outcomes after self-harm, standard activities of daily living or disability measures are too oriented to physical deficits, although quite practical activities are an obvious challenge for some, such as the ability to use public transport or to manage paid employment. Measures of more social function exist for use in disability research^12^ but an important challenge remains the lack of measures of social function that are comprehensive, cover both practical activities and subjective sense of participation or belonging, and are entirely suitable for a population as heterogeneous as those recruited into self-harm trials.

## Achievable outcomes measurement

In this context achievability means two things: the ability to identify and use individual measures with acceptable properties; and the ability to use those measures with an evaluation design that takes into account the various outcome domains and the heterogeneity of the target population.

### Characteristics of an individual measure

Of course, any measure should have good psychometric properties, including sensitivity to change. This latter is especially a challenge for measures of social participation because of the slowness of achieving meaningful change, which entails both increasing the physical actions entailed and also the sense of meaningfulness or satisfaction from those actions. For example, it is possible to reduce the amount of time a person spends alone without reducing their sense of loneliness. The contingencies of research impose limits here as outcomes need to be measurable on an affordable (for research funders) timescale that rarely extends beyond 12 months when the measure requires contact with participants, as opposed to use of routine data.

A second consideration is that outcome measure should be, at least potentially, applicable to most or all of the self-harm population and should be seen as relevant by them. This last feature can create problems. For example, people with a history of repeated self-harm may not, as noted earlier, see short-term reduction in repetition as an outcome that is relevant to them compared with, say, an improvement their sense of self-worth and ability to feel comfortable in relationships with others. By the same token, engagement with mental health services, as suggested by Owens and colleagues, is an important prerequisite for obtaining benefit from formal therapeutic approaches but would not be endorsed by all as an important outcome.

### Responding to the challenges: multiple outcome domains and population heterogeneity

Another problem when it comes to responding to these challenges resides in the constraints imposed by conventional RCT design. The RCT typically requires a single primary outcome upon which sample size calculations are based, including where appropriate estimates of clustering for example by therapist.

The use of more than one primary outcome is not impossible, but multiple outcomes raise formidable analytic problems^13^ even if researchers can overcome the practical challenge of low completion rates as a result of respondent burden. Composite measures have been used, but only when the elements are meaningfully related to each other – suicidal thinking, non-fatal self-harm and suicide for example – but even then they are problematic. There is no plausible composite outcome that could be derived from the diverse outcomes outlined above.

Is personalisation possible? That is, could outcome for each participant be individual to that person, with grouping of results achieved by standardising the response format? One example is Goal Attainment Scaling.^14^ Although appealing in principle, concerns about reliability of scores and of their comparability across individuals and groups of individuals has limited the take up of such approaches in RCTs.

## Conclusions

Owens and colleagues are right to remind us that research into intervention after self-harm has tended to overemphasise repetition at the expense of other outcomes that are important to study participants. Where they are wrong is in attributing the problem to lack of understanding of the nature of self-harm: a more cogent criticism is that we have lacked imagination in thinking about how to overcome the constraints imposed by conventional RCT design.

There are two immediate remedies. One is that if we are sticking with conventional RCTs as our preferred evaluation design then we should consider other primary outcomes. For example in our own currently funded trial with participants who have a history of repeated self-harm the primary outcome is quality of life. An alternative is to branch out in our use of other designs – modelling observational data and exploiting the potential that comes from linking observational data to RCT data, combining the benefit of RCTs, especially in dealing with confounding by indication, with the greater capacity of observational data to encompass heterogeneity.^15^
